# Effects of Cinchona and the Sulphate of Quinia

**Published:** 1845-05

**Authors:** 


*Effects of Cinchona and the Sulphate of Quinia.*—M. Felix Jacquot, principal of the medical clinic of the military hospital of instruction at Metz, has a long paper in the *Archiv. Gén. de Méd.*, September, 1844, on the relative operation of Peruvian bark, and its chief alkaloid in various diseases. There is no novelty, assuredly, in his telling us that the bark is a powerful tonic and a good febrifuge; but when he adds, that quinia and its salts are eminently febrifuge but not tonic, or if so, in a very limited degree, his views will sound odd to many who have not analyzed the phenomena of fevers, and noted the effects of different classes of medicines on these diseases, carefully separating what is really tonic in their operation, from that which is sedative. In the cinchonic bark there are, as M. Jacquot points out, but not for the first time, two elements, the tonic, and the modifier of the nervous system. The latter is quinia, while the former is made up of the union of several principles, but especially by the cinchonic bitter of Reuss, who regarded this substance as the active principle.

The most accredited succedanea for cinchona, regarded as a febrifuge, are tonics and sedatives. Of the former, the list is very large, and includes chalybeates, various vegetable bitters, and astringents, to which may be added substantial nourishment. The operation of this class, as febrifuges, is slow and gradual; but on account of this slowness of effects, they cannot be relied on to cut short fevers at once, as quinia does. They are adapted to debility, anemia, and other sequelæ of protracted fever. They complete what quinia had begun. To this latter we have recourse in all paroxysmal attacks with recurrence, in nervous complications, as in congestive fevers or pernicious intermittents, and in plethoric states in which a tonic is not demanded. Cinchona fulfils, therefore, other indications than quinia, being both a specific febrifuge in consequence of this latter principle, and a general tonic owing to its bitter, and most probably other principles. This opinion, which might be inferred from the chemical composition of the Peruvian bark, independently or in advance of clinical trials, is probably the true one, but which, of late years, seems to be forgotten or neglected in the treatment of our periodical fevers.

M. Jacquot declares his belief that quinia is a sedative of the nervous system; and probably of the sanguiferous system also. In support of this view he refers to the most approved substitutes for quinia, independently of the tonic class, such as various narcotics, the combinations of cyanid acid with different bases, and such sedatives as tartar emetic, &c.; and next, he directs attention to the physiological effects of quinia in large doses, as being sedative, and to the practitioners who daily employ quinia as a medicine of this nature.

We may be supposed to accord our assent to these views of M. Jacquot, as we have for some years past entertained and publicly expressed similar ones. In one of our lectures on *congestive fever*, in the first edition of Stokes and Bell's Lectures, (1840) we used the following language: "You will give the quinia with more freedom when you give it a as a sedative, or a means at any rate, if this term sounds exceptionable, of removing entirely the irritation which originated and kept up the paroxysm." "The operation of quinine is not antagonistic to that of blood-letting, nor is it congenial with inflammation." In speaking of dose we remarked: "If we are desirous of making an impression at all decided on the nervous system, and through its sedation of allaying the febrile disturbance of the functions generally, five grains of the sulphate of quinine is the smallest dose which we should think of prescribing for an adult whose idiosyncrasy is not such as to forbid the use of the medicine beyond minute doses."

Since this opinion was advanced in favor of the sedative operation of quinia, farther reflection and observation strengthen our convictions of its accuracy; and some European writers and practitioners are now, like M. Jacquot, distinctly advocating the same view.

In typhoid fever M. Jacquot thinks favorably of quinia; not as a means of arresting the disease, but destroying its diurnal paroxysms, and other epiphenomena which give it an ataxic character, and the removal of which simplifies it to a great extent.—*Bulletin of Medical Science.*

